# Combining Radiation Therapy with Immune Checkpoint Blockade for Central Nervous System Malignancies

**DOI:** 10.3389/fonc.2016.00212

**Published:** 2016-10-07

**Authors:** Neil M. D’Souza, Penny Fang, Jennifer Logan, Jinzhong Yang, Wen Jiang, Jing Li

**Affiliations:** ^1^Department of Radiation Oncology, The University of Texas MD Anderson Cancer Center, Houston, TX, USA; ^2^Baylor College of Medicine, Houston, TX, USA

**Keywords:** immune checkpoint blockade, immunotherapy, radiotherapy, CTLA-4, PD-1, glioblastoma, brain metastases, brain neoplasms

## Abstract

Malignancies of the central nervous system (CNS), particularly glioblastoma and brain metastases from a variety of disease sites, are difficult to treat despite advances in multimodality approaches consisting of surgery, chemotherapy, and radiation therapy (RT). Recent successes of immunotherapeutic strategies including immune checkpoint blockade (ICB) *via* anti-PD-1 and anti-CTLA-4 antibodies against aggressive cancers, such as melanoma, non-small cell lung cancer, and renal cell carcinoma, have presented an exciting opportunity to translate these strategies for CNS malignancies. Moreover, *via* both localized cytotoxicity and systemic proinflammatory effects, the role of RT in enhancing antitumor immune response and, therefore, promoting tumor control is being re-examined, with several preclinical and clinical studies demonstrating potential synergistic effect of RT with ICB in the treatment of primary and metastatic CNS tumors. In this review, we highlight the preclinical evidence supporting the immunomodulatory effect of RT and discuss the rationales for its combination with ICB to promote antitumor immune response. We then outline the current clinical experience of combining RT with ICB in the treatment of multiple primary and metastatic brain tumors. Finally, we review advances in characterizing and modifying tumor radioimmunotherapy responses using biomarkers and microRNA (miRNA) that may potentially be used to guide clinical decision-making in the near future.

## Introduction

Recent advances in the field of tumor immunology, such as the discovery of monoclonal antibodies targeting immune checkpoints, have opened a new frontier in the fight against cancer. These monoclonal antibodies recognize and inhibit immune suppressive functions mediated by cytotoxic T-lymphocyte associated protein 4 (CTLA-4) and programmed cell death protein 1 (PD-1) receptors to promote immune-mediated antitumor activity (1–5). In the setting of metastatic melanoma, non-small cell lung cancer (NSCLC), Hodgkin’s lymphoma, and renal cell carcinoma (RCC), these agents achieved significant improvement in clinical responses and survival as monotherapies, but only in limited subsets of patients (1, 6–8). In order to broaden clinical utility and efficacy, recent investigations have focused on combination schemes that provide immune activation, while counteracting inhibitory checkpoint signals. Radiation therapy (RT) has traditionally been perceived to be a local treatment strategy with potentially immunosuppressive effects (1, 9). However, recent data suggests that RT can trigger the “abscopal effect,” which describes treatment response in tumors outside the radiation fields through systemic immune-mediated antitumor effects (1, 10–14). Other types of immunostimulatory effects of RT have been observed in preclinical studies, and recent clinical studies exploring the role of RT in combination with immunotherapy have shown synergism between these two treatment modalities (15–17).

The brain has traditionally been considered an immunoprivileged entity with most systemic therapies demonstrating minimal to no intracranial activity. While recent advances in systemic therapy have significantly improved patient outcomes including survival, treatment of brain metastasis continues to represent a unique challenge, particularly for patients with neurotropic primaries, such as melanoma, NSCLC, and breast cancer (18). Similar challenges exist in the treatment of primary central nervous system (CNS) malignancies, such as glioblastoma, which is highly invasive and carries a 5-year survival rate of less than 10% (19, 20).

Fortunately, recent data have suggested that the CNS does indeed interact with the immune system and is subject to a dynamic regulation *via* proinflammatory and immunosuppressive forces (21–23). With the development of immune checkpoint blockade (ICB), studies investigating combination therapy of ICB with traditional standard treatment, including RT, have suggested potential synergistic effects in the brain (24–27). In this review, we provide an overview of the immune modulatory effect of RT and rationales for radioimmunotherapy using ICB. We also aim to explore the future outlook of this emerging paradigm as well as the development of new biomarker platforms that can help harness the full potential of this combined approach in the treatment of CNS malignancies.

## Preclinical Rationales

### Immunostimulatory and Systemic Antitumor Effects of Radiation Therapy

Although radiation has traditionally been considered an immunologically inert process, the recent discovery of immunogenic cell death (ICD), a unique mode of cell death induced by RT or chemotherapy *via* potent host-mediated antitumor response (28), has suggested otherwise. Cell death occurs differently depending on the identity and maturity of the phagocytic cell, location and manner of phagocytosis, the availability of helper T-lymphocytes, type of death pathway that is triggered, release of immunosuppressive mediators (TGF-β, IL-10), and the immune cells that are exposed to antigens (29). ICD in particular is primarily defined by unique molecular processes, including the translocation of calreticulin (CRT) to the cell surface, ATP release, upregulation of costimulatory molecules, and the extracellular release of high-mobility group protein B1 (HMG-B1), which enhances antigen cross-presentation and secretion of proinflammatory cytokines (28, 30).

**Figure 1:**
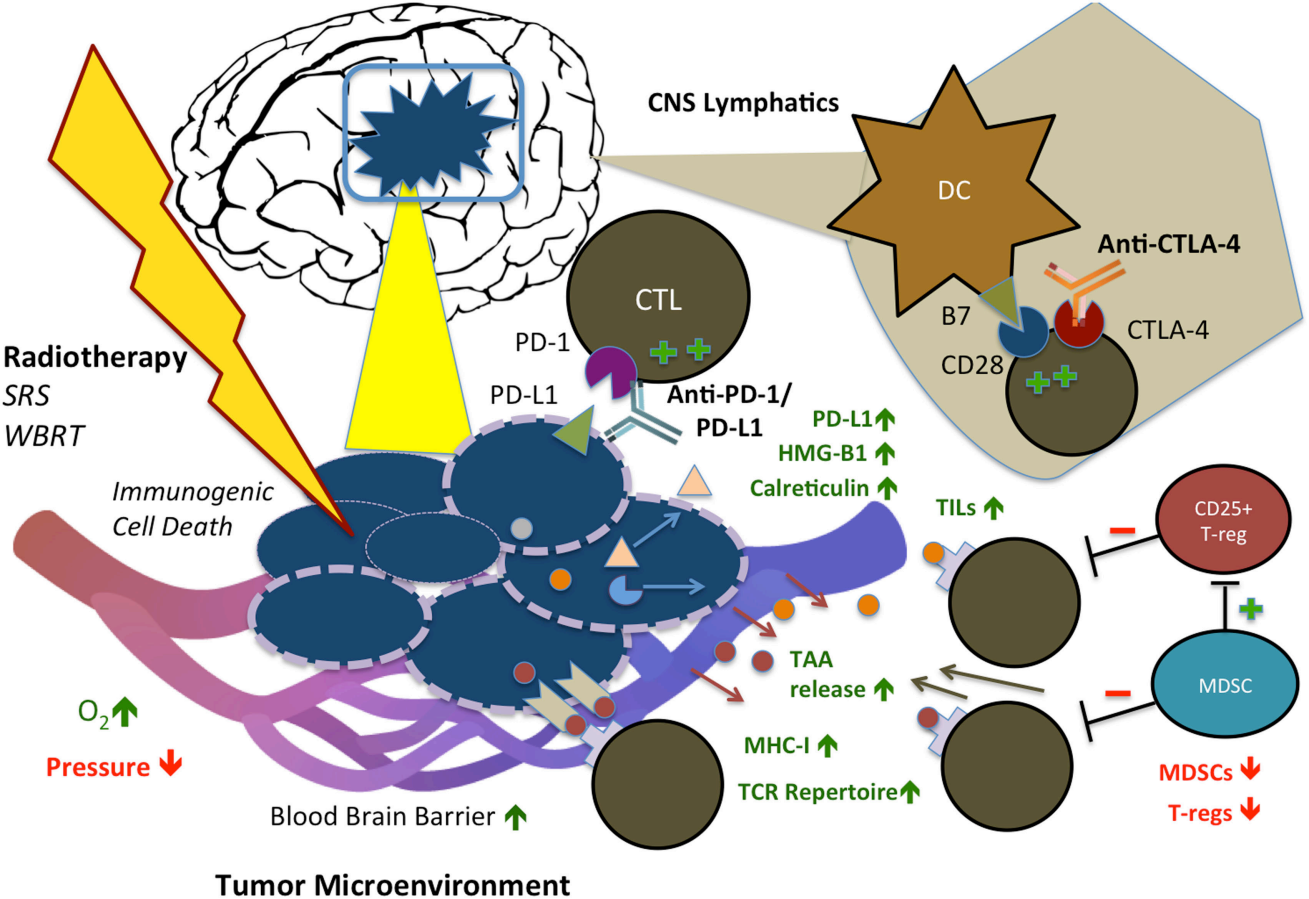
**Immunostimulatory effects of radiation therapy (RT) in combination with immune checkpoint blockade (ICB) in the CNS**. RT and ICB work synergistically to create an immunogenic tumor microenvironment and promote systemic antitumor response. Anti-PD-1 and PD-L1 agents reduce tumor cell-mediated exhaustion signals to CD8^+^ CTLs, while anti-CTLA-4 agents block competing co-inhibitory activity of CTLA-4, resulting in increased and persistent T-cell activation. RT triggers immunogenic cell death (ICD) of tumor cells, displacement of calreticulin (CRT) to the cell surface, release of HMG-B1, increased MHC-I expression, and release of tumor-associated antigens (TAAs), with consequent increase in TCR repertoire of infiltrating T-cells. In addition, RT has stromal effects on the tumor microenvironment: increasing oxygenation, infiltration of TILs, and permeability of the blood–brain barrier (BBB), while decreasing interstitial fluid pressure. In combination with ICB, RT also reduces activity of T-regs and MDSCs. SRS, stereotactic radiosurgery; WBRT, whole brain radiation therapy; CTL, cytotoxic T-lymphocyte; PD-1/L1, programmed cell death protein 1/ligand 1; CTLA-4, cytotoxic T-lymphocyte-associated protein 4; DC, dendritic cell; TIL, tumor-infiltrating lymphocyte; TAA, tumor-associated antigen; MHC, major histocompatibility complex; TCR, T-cell receptor; MDSC, myeloid-derived suppressor cell; HMG-B1, high mobility group box 1.

Although RT has been speculated to exert immunosuppressive effects *via* increased TGF-beta expression, M2 macrophage polarization, and T-regulatory (T-reg) cell recruitment, its immunostimulatory effect is beginning to be understood (31–34). Ionizing radiation has been shown to increase translocation and expression of CRT (35) and promote gene transcription of proinflammatory factors *via* HMG-B1 (36, 37), which are the essential components of ICD, as well as reduce production of immune suppressive cytokines and increase expression of MHC-I and synthesis of novel peptides for cytotoxic T cell recognition (38). Moreover, RT has been shown to promote re-oxygenation and decrease interstitial fluid pressure within the tumor microenvironment, improving immune cell recruitment and infiltration into irradiated tumor (39). Finally, RT induces the release of tumor-associated antigens (TAAs), diversifying the TCR (T-cell receptor) repertoire of infiltrating CTLs and leading to increased efficacy of CTLs (27, 40). Notably, these cancer-specific and stromal-associated responses occur simultaneously and define radiation-induced immunogenicity of the tumor cells. Preclinical studies have clearly suggested that radiation, although conventionally perceived as a local therapy, can potentially exert systemic antitumor effects at least through both cancer cell intrinsic and tumor microenvironmental modulations. These mechanisms are illustrated in Figure 1.

### Immune Checkpoint Blockade and CNS Tumors

While the brain has traditionally been considered an immunoprivileged organ system, it is now commonly accepted that dysregulation of immune surveillance may be involved in the pathogenesis and progression of multiple CNS malignancies (41). Similar to other types of cancers, primary brain tumors can evade immune system detection through multiple immunosuppressive mechanisms, including upregulation of immune checkpoints. For example, PD-L1 has been shown to be upregulated in glioblastoma cells as well as circulating monocytes and macrophages through oncogenic signaling resulting from PTEN loss and modulation of autocrine–paracrine IL-10 signaling (42–44). Increased PD-1 and PD-L1 expression has also been observed in brain metastases and primary CNS lymphomas (45, 46). These findings warrant further exploration of ICB, in particular PD-1 or PD-L1 blockers, in the pathogenesis and treatment of primary and metastatic CNS malignancies.

Radiation therapy is the pillar of standard therapy for primary and secondary brain tumors. The immunostimulatory effects of RT make it a natural candidate as a synergistic partner with ICB. Indeed, prior preclinical studies have shown survival benefits of radiation with tumor vaccination in glioma mice models (20). In melanoma, RT has been shown to diversify TCR repertoire of infiltrating CTLs and enhance their function *via* RT-induced release of TAAs (27). This mechanism worked in concert with an anti-CTLA-4 agent leading to inhibition of T-reg cell function and increased CD8^+^ T cell/T-reg ratio. Interestingly, the addition of an anti-PD-L1 agent further prevented T cell exhaustion (40). In murine glioma models, combining radiation concurrently with anti-PD-1 and anti-CTLA-4 or 4-1BB (a costimulatory molecule) agonist yielded improved survival and increased tumor-infiltrating leukocyte (TIL) population, compared with either modality alone (24, 47). The rationale for combining RT with PD-1/PD-L1 blockers is further supported by the observation that local inflammation mediated by RT results in PD-L1 upregulation on cancer cells, macrophages, and dendritic cells (DCs) (48). Moreover, a recent study in murine melanoma brain metastases (MBM) showed that increasing radiation dose with concurrent immunotherapy improved median and long-term survival and prolonged tumor dormancy (49). Finally, while the penetrance of ICB agents into the CNS *via* the blood–brain barrier (BBB) is not fully known, RT has been shown to increase BBB permeability (50), further facilitating the penetrance of activated antitumor immune cells and possibly the access of ICB agents as well – providing another compelling rationale for combination RT-ICB therapy in the treatment of primary and metastatic CNS tumors (Figure 1).

## Clinical Studies and Experience

The combination of RT and ICB has been reported to improve clinical outcomes in multiple metastatic cancers. In metastatic melanoma, potential radiation-induced abscopal responses have been reported in the setting of CTLA-4 blockade (10, 51, 52). The first of such reports was by Postow et al., who described a patient with paraspinal, splenic, and right hilar metastatic disease receiving palliative radiation to the paraspinal lesion with concurrent ipilimumab. Five months after treatment, all lesions demonstrated regression on restaging CT scans (10). Similar case studies have reported potential clinical benefits of combining radiation with ICB in metastatic melanoma (51, 52) as well as NSCLC (53).

In addition to immunogenic tumors, such as melanoma and NSCLC, the role of radiation combined with ICB has also been investigated in metastatic, castration-resistant prostate cancer. A recently completed phase I/II trial looked at combining ipilimumab and palliative RT to bony disease and found a complete response rate of 2% and stable disease in 12% of patients (54). Although no difference in overall survival was noted, there were signs of enhanced antitumor activities in the ipilimumab arm with decreased PSA and improved progression-free survival (PFS). It is, however, questionable, whether radiation contributed to these benefits as both the ipilimumab and placebo arms received RT. Another early trial attempted to ask this question by looking at ipilimumab vs. ipilimumab plus RT in metastatic prostate cancer and demonstrated promising clinical response (55). However, a phase III trial of this regimen in men with previously treated CRPC failed to demonstrate a significant overall survival benefit (56).

In the metastatic disease setting, there are multiple ongoing trials combining RT, particularly stereotactic radiosurgery (SRS) or hypofractionated radiation, with immunotherapy in multiple cancer types. At MD Anderson, we are currently investigating different combinations of immune checkpoint blockers with stereotactic body radiation therapy (SBRT) for lung and liver metastasis from multiple primary sites (NCT02239900, NCT02402920, NCT02444741).

### Brain Metastases

Clinical evidence supporting the efficacy of combining RT and ICB in the treatment of CNS tumors is primarily garnered from melanoma patients with metastatic disease and is retrospective in nature. Three retrospective single-institution studies have suggested that ipilimumab in combination with RT may be more effective than RT alone in MBM (57–60). Knisely et al. reviewed the outcomes of 77 patients with MBM who received SRS as well as ipilimumab. Patients who received combination therapy demonstrated improved survival compared with those who received SRS alone (58). Moreover, a study by Silk et al. explored the benefits of combination therapy vs. RT alone in 70 MBM patients who received brain radiation (either SRS or WBRT) with ipilimumab. Retrospective analysis demonstrated an OS benefit of 19.9 months with combination therapy vs. 4.0 months for SRS alone, with no associated increase in toxicity with addition of ipilimumab to SRS (59). An interesting study by Kiess et al. investigated the impact of timing in administration of SRS for brain metastases and ICB on patient outcome. Patients who received SRS before or concurrently with ipilimumab appeared to exhibit improved outcomes in OS and distant intracranial control compared to patients who received radiation after immunotherapy (60). Notably, responses to ipilimumab therapy in the brain, as in extracranial disease, can be quite durable with some reported response beyond 4 years (61). Given the retrospective and single institutional nature of these studies, caution needs to be taken regarding interpretation of their results. Several prospective early phase clinical trials at multiple institutions are investigating different combination regimens of RT with immune checkpoint blockers in the setting of brain metastases (Table 1). Results from these studies will one day shed light on the clinical benefit of combining radiation with immunotherapy for the treatment of brain metastases.

**Table 1:** **Current clinical trials of immunotherapy with radiation for primary and metastatic CNS malignancy**.

Study phase	Institution/group	ClinicalTrials.gov ID	Disease site	Cohorts	Planned accrual	IT mechanism	Est. completion date	Primary outcome measure
II	Multi-institutional (CheckMate548)	NCT02667587	Newly diagnosed glioblastoma	Nivolumab + temozolomide + RT vs. placebo + temozolomide + RT	*n* = 320	anti-PD-1	May 2017	OS
III	Multi-institutional (CheckMate498)	NCT02617589	Newly diagnosed glioblastoma	Nivolumab + RT vs. temozolomide + RT	*n* = 550	anti-PD-1	October 2019	OS
II	Ludwig Institute for Cancer Research	NCT02336165	Newly diagnosed, recurrent glioblastoma	MEDI4736 vs. MEDI4736 + standard RT vs. MEDI4736 + bevacizumab	*n* = 108	anti-PD-1	July 2017	OS, PFS
I/II	Northwestern University	NCT02530502	Newly diagnosed glioblastoma	RT + temozolomide + pembrolizumab → temozolomide + pembrolizumab	*n* = 50	anti-PD-1	March 2018	Dosage, PFS, OS
I	H. Lee Moffitt Cancer Center	NCT02313272	Recurrent glioma	HFSRT + pembrolizumab + bevacizumab	*n* = 32	anti-PD-1	June 2017	Dosage
I/II	MD Anderson Cancer Center	NCT02696993	NSCLC BM	Nivolumab + SRS; nivolumab + WBRT; nivolumab + ipilimumab + SRS; nivolumab + ipilimumab + WBRT	*n* = 130	anti-PD-1; anti-CTLA-4	April 2020	Dosage; PFS
II	Grupo Español Multidisciplinar de Melanoma (GEM)	NCT02115139	Melanoma BM	Ipilimumab + WBRT	*n* = 66	anti-CTLA-4	October 2016	1-year survival rate
II	University of Michigan Cancer Center	NCT02097732	Melanoma BM	Ipilimumab → SRS → ipilimumab vs. SRS → ipilimumab	*n* = 40	anti-CTLA-4	May 2017	Local control rate
I	Thomas Jefferson University	NCT01703507	Melanoma BM	Ipilimumab + WBRT vs. ipilimumab + SRS	*n* = 24	anti-CTLA-4	November 2017	Dosage
I	Sidney Kimmel Comprehensive Cancer Center	NCT01950195	Melanoma BM	Ipilimumab + SRS	*n* = 30	anti-CTLA-4	December 2016	Adverse events and safety
II	University Hospital, Lille	NCT02662725	Melanoma BM	Ipilimumab + SRS	*n* = 73	anti-CTLA-4	December 2015	OS

*RT, radiation therapy; PD-1, programmed cell death protein 1; OS overall survival, PFS, progression-free survival; HFSRT, hypofractionated stereotactic radiotherapy; IMRT, intensity-modulated radiation therapy; CTLA-4, cytotoxic T-lymphocyte-associated protein 4; irRC, immune-related response criteria; WBRT, whole brain radiation therapy; NSCLC, non-small cell lung cancer; BM, brain metastases; SRS, stereotactic radiosurgery; MM, metastatic melanoma; SBRT, stereotactic body radiation therapy*.

### Primary Brain Tumors and Ongoing Clinical Trials

For primary CNS tumors, such as glioblastoma, several combination approaches with radiation have been explored clinically (62). For example, immunotherapy in the form of vaccines derived from autologous DCs and pulsed with tumor lysate and various tumor antigens (such as the EGFRvIII vaccine Rindopepimut) have shown some promise in the clinical setting (20). A randomized phase II trial testing ICT-107, a vaccine composed of autologous DCs and tumor antigens, administered with radiation and temozolomide for newly diagnosed glioblastoma, demonstrated benefit in terms of OS and PFS for a subset of HLA-A2 positive patients (63).

Although evidence supporting the combination of ICB and RT for glioblastoma is currently lacking, several clinical trials are underway examining this combination for newly diagnosed and recurrent glioblastoma (Table 1). Two of these are large multi-institutional trials in phases II and III (NCT02667587, NCT02617589) examining OS in patients given nivolumab in combination with radiation (with/without concurrent temozolomide) for newly diagnosed glioblastoma, while another smaller trial is examining similar combinations with pembrolizumab (NCT02530502). A phase II trial sponsored by the Ludwig Institute is investigating OS in monotherapy with the PD-1 inhibitor durvalumab (MEDI4736) in comparison to combination approaches with standard RT or the VEGF inhibitor bevacizumab. Finally, a phase I trial is examining maximum dosage of pembrolizumab when it is used with combination therapy with bevacizumab and a hypofractionated radiation regimen given over 5 days for recurrent glioma. Additional clinical trials investigating combination approaches in the context of primary CNS malignancy will soon be underway.

### Future Outlook

Glioblastoma is notorious for employing a wide variety of immunosuppressive strategies to disrupt the function of immune cells, lower immunoglobulin levels, and generate a plethora of immunosuppressive processes, involving TGF-beta, IL-10, MHC-I downregulation, T-regs recruitment, and increased expression of PD-L1 (20, 64–68). Development of therapeutics for glioblastoma is complicated by the variability in antigen expression within individual tumors and subtypes. The ability to target the unique signature of tumor-specific mutations and antigens (“neoantigens”) is crucial in effective tumor control, and the expression of these neoantigens has been shown to predict long-term response to ICB in metastatic melanoma and NSCLC (69, 70). Strategies such as biomarker and microRNA (miRNA) development are under investigation to predict and modulate tumor response to immunotherapy and RT.

### Biomarker Identification

Along with clinical trials, current translational research efforts are exploring potential predictive biomarkers for response to immunotherapies in glioblastoma (71). Increased PD-L1 expression in circulating monocytes has been identified as a biomarker for tumor-induced immunosuppression and a prognostic factor for poor survival. For example, in one study of glioblastoma patients, the median overall survival for high-expressers of PD-L1 was 18.0 months compared with 44.7 months for low PD-L1 peripheral monocyte expressers (5). Another example is level of tumor EGFRvIII expression as a biomarker for response to the EGFRvIII vaccine in glioblastoma (72). Increased peripheral blood levels of CD33^+^HLA-DR^−^ myeloid-derived suppressor cells (MDSCs), with the potential to induce T-regs and apoptosis in activated lymphocytes as well as secrete immunosuppressive cytokines and oxidizing molecules, have also been reported patients with glioblastoma. RT in combination with ICB has been shown to reduce tumor-associated MDSCs in comparison to single therapy *via* the activity of CD8^+^ T cells and tumor necrosis factor (TNF) signaling *in vivo* (48). Moreover, a diminution in the level of circulating T-regs has shown promise as a biomarker for treatment response to immunotherapies (73).

MicroRNAs can act as oncogenes or tumor suppressors through controlling target gene expression and binding of mRNA, and also carry promise as biomarkers (74). In glioblastoma, tumor miR-21 has been a promising candidate as a biomarker in diagnosis and response to chemotherapies (75). In the serum, levels of miR-128, miR-320, and miR-574-3p have been found to be elevated (76, 77). Interestingly, the presence of metastatic disease in the brain can be distinguished from glioblastoma *via* the identification of the miR-200 family in cerebrospinal fluid (78). miRNA can also serve as targets for therapy. Double-stranded miRNA mimics can reinstate activity of tumor suppressor miRNA, and oncogenic miRNAs can be disenabled by single-stranded antisense nucleotides known as “antimiRs” (79). One example in the CNS is miR-296, which led to reduced tumor volume and angiogenesis in a murine glioma model (80). Another miRNA, miR-221/222, demonstrated reduced tumor volume in murine melanoma (79). A potential target for an miRNA mimic in ICB is miR-138, which has been demonstrated to target both CTLA-4 and PD-1, inhibiting tumor-infiltrating T-regs and subsequently releasing the brake set by these immunosuppressive cells within the glioblastoma microenvironment (81). Whether specific miRNA can be used to predict treatment responses to immunotherapy warrants further investigation.

Newer techniques, such as multi-parameter flow cytometry, time-of-flight mass cytometry (CyTOF) and whole-repertoire T and B receptor amplification, can be used to characterize the immunologic cellular makeup of primary and metastatic brain lesions at baseline and changes in circulatory immune cell profile during treatment (1, 72, 82, 83). RNAseq transcriptome profiling of tumor samples can quantify the presence of markers, tumor antigens, and certain genes. The latter has demonstrated utility in predicting survival in neuroblastoma and treatment response in breast cancer (84). Finally, “imaging genomics” or “radiomic” analysis may be a cost-effective and non-invasive strategy to link the radiophenotype of a tumor it its underlying genomic and proteomic characteristics, *via* the high-throughput extraction and analysis of large amounts of radiographic imaging for distinctive features, which can then be mapped to certain genomic and proteomic patterns such that predictive and prognostic classifications may be made (85–88). Radiomic analysis may be particularly useful to characterize and distinguish radiation necrosis (RN), a potential late adverse effect of high-dose focal irradiation to the brain occurring more frequently in patients receiving ICB (89), from tumor progression or recurrence and the phenomenon of pseudoprogression. Furthermore, this strategy may be used with traditionally measured biomarkers and miRNA profiles to construct multi-dimensional schemes for prediction of response to combining ICB and RT, development of tailored treatment plans, and assessment of adverse effects of such therapy.

## Conclusion

A new generation of immunotherapeutics, most prominently the immune checkpoint inhibitors, has ushered in a new era of cancer treatment strategies. Despite the effectiveness of these agents, significant limitations remain. Multiple studies have begun to reveal a synergistic effect between ICB and RT, with numerous clinical trials exploring combinatorial strategies in diverse sites of disease. Primary CNS malignancies, such as glioblastoma, and far more commonly metastatic lesions, carry a grim prognosis and stand to gain much benefit from novel combination approaches utilizing RT and ICB. Benefits in terms of survival and intracranial control have been shown in the preclinical setting and are now being tested in various trials. However, many important questions remain to be answered. Nevertheless, with improved understanding of how RT interacts with the immune system, combined strategies that utilize RT with ICB may provide a new and effective to treat CNS malignancies.

## Author Contributions

NMD performed the literature research. All authors helped to write the manuscript.

## Conflict of Interest Statement

JLi received research funding from BMS to support the research expenses of a phase I/II clinical trial with immunotherapy. The rest of the authors report no conflicts of interest.
